# Metastasis from small cell lung cancer to ovary: A case report

**DOI:** 10.1515/biol-2022-0684

**Published:** 2023-09-01

**Authors:** Benzheng Zhao, Weixin Zhao, Yang Xia, Jian Liu, Yuan Wang, Tianjiao Song, Wenxi Tan, Shuhua Zhao

**Affiliations:** Department of Obstetrics and Gynecology, Second Hospital of Jilin University, Changchun 130041, China; Department of Pathology, Second Hospital of Jilin University, Changchun 130041, China; School of Nursing, Jilin University, Changchun 130021, China

**Keywords:** small cell cancer, metastatic ovarian tumor, hypercalcemic type, pulmonary type, case report

## Abstract

Small cell lung cancer (SCLC) rarely metastasizes to the ovary, and is difficult to diagnose given its overlapping clinical features and histological characteristics with primary ovarian cancer. Since therapies for SCLC and primary ovarian cancer differ, it is important to determine the original site of ovarian lesions. This report describes the differential diagnosis of metastatic from primary ovarian cancer. A 46-year-old Chinese woman with a history of SCLC, confirmed by transbronchial lung biopsy in August 2018, presented with abdominal distension in December 2018. Ultrasound examination and whole abdomen computed tomography showed one mass in each ovary. A provisional diagnosis of ovarian tumor was given. A palliative total abdominal hysterectomy and bilateral salpingo-oophorectomy was performed; and three postoperative courses of chemotherapies. The patient died from multiple organ failure in May 2019. Metastatic ovarian cancer from SCLC was determined based on characteristic histological and immunohistochemical staining.

## 1 Introduction

Small cell tumors originate in the neuroendocrine system and are commonly found in the lungs and are known as small cell lung cancer (SCLC). SCLC has a significant tendency to metastasize, usually occurring early in the disease [1]. Metastatic disease usually coincides with the first diagnosis of the primary tumor. Common metastatic sites of SCLC are brain, bone, and liver. Metastasis to other organs is relatively rare. SCLC metastasis to the ovary is diffuse, and only a very small number of such cases have been reported in our extensive literature search [2]. Uncommon metastases not only affect diagnosis of the disease, but also, importantly, often indicate a poor prognosis. The discussion of domestic and foreign scholars on ovarian SCLC mainly focuses on the pathological features, diagnosis, and differential diagnosis of two types (SCCOHT and SCCOPT) of primary small cell ovarian cancer (SCCO), but in any case, they will eventually receive similar treatment. It is worth noting that metastasis SCCO has a worse prognosis than SCCO, and metastasis SCCO is different from SCCOPT when chemotherapy or radiotherapy is given after surgery according to the nature of small cell lung tumor, so it is necessary to distinguish in diagnosis. However, the histological features of metastasis SCCO and SCCOPT are similar, so it is difficult to distinguish them in clinicopathology. In this study, immunohistochemical staining was applied to differentiate SCLC metastasis from primary ovarian tumor and accurately assess the disease and prognosis of ovarian cancer patients. Following breakthroughs in immunotherapy and targeted therapy in recent years [3,4], we reviewed the case in the context of new therapeutic advances, publicizing treatment regimens, pathological types, and immunohistochemical data. We hope that by collating high-quality clinical and epidemiological data will give us a better understanding of the disease, encourage the combined use of antiangiogenic agents or other targeted agents in future disease treatment, lead to the development of effective immunotherapy or antibody coupling agents, and act as catalysts for improving overall patient outcomes.

The main histological types of ovarian malignancies include ovarian epithelial tumor, ovarian germ cell tumor, ovarian sex cord stromal tumor, and ovarian metastatic tumor (OMT). The most common type of OMT is Krukenberg tumor, which has a primary origin in the gastrointestinal tract. The principle of treatment for OMTs is to relieve and control symptoms. If the primary tumor has been removed and there are no other signs of metastasis and the metastatic tumor is confined to the pelvic cavity, complete hysterectomy and bilateral adnexectomy should be performed, and the pelvic metastasis should be removed as far as possible. Chemotherapy or radiotherapy should be given after surgery depending on the nature of the primary tumor. The vast majority of OMTs have poor therapeutic effect and poor prognosis, and most of them die within 2 years.

Ovarian SCLC includes both primary and metastatic tumors. There are two types of ovarian primary small cell lung cancer (SCCO): the hypercalcemic type (SCCOHT) and the pulmonary type (SCCOPT). SCCOHT is more common than SCCOPT and occurs in young women with elevated serum calcium levels, which may be genetically related. However, SCCOPT often occurs in older women and is sensitive to chemotherapy [2]. Primary SCCO may be classified as either hypercalcemic type (SCCOHT) or pulmonary type (SCCOPT). The true origins of either remain controversial. Jan et al. [5] proposed that primary SCLC originates in ovarian epithelium, while according to Ulbright et al., it originates from germ cells. The treatment methods of both are mainly surgery, adjuvant chemotherapy, and comprehensive treatment.

## 2 Case presentation

A 46-year-old Chinese woman, a non-smoker, was admitted to Second Hospital of Jilin University in December 2018, due to abdominal distension. She had a history of SCLC from August 2018, with computed tomography (CT) scanning showing a mass in the upper lobe in the right lung. Subsequently, SCLC was confirmed by transbronchial lung biopsy.

The immunohistochemical staining showed the following: chromogranin A (CgA) (–), synaptophysin (+), neural cell adhesion molecule 56 (CD56) (+), Ki67 (60%), cytokeratin AE1/AE3 (focal positive), thyroid transcription factor 1 (TTF-1) (+), and leukocyte common antigen (+). Serum tumor markers revealed CA125, 44.90 U/mL (normal 0–35 U/mL); CA199, 10.74 U/mL (normal 0–37 U/mL); and neuron-specific enolase, 24.70 ng/mL (normal 0–15 ng/mL). Radio chemotherapy was implemented to control tumors.

**Figure 1:**
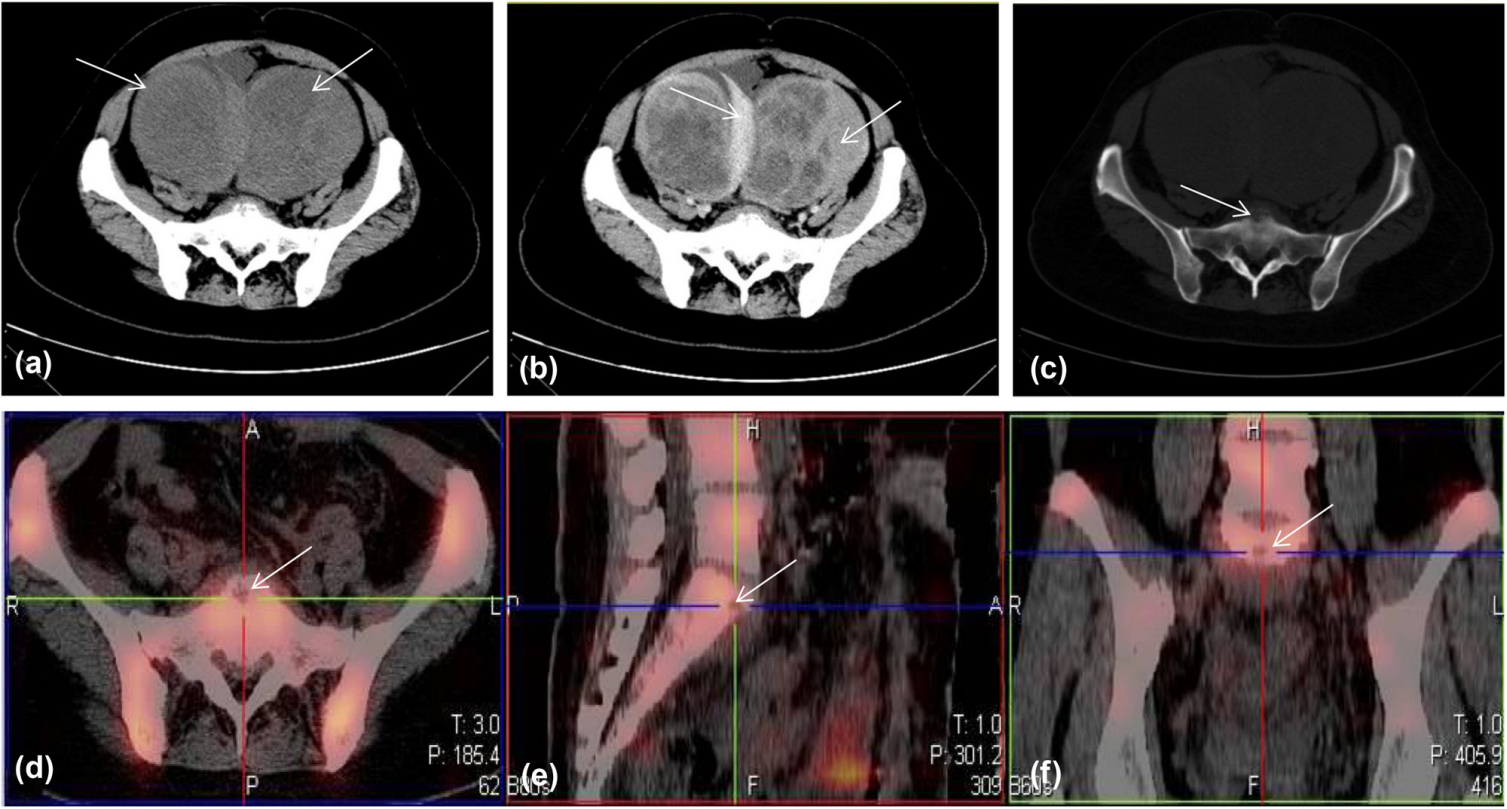
Whole abdomen CT (a–c) and radionuclide bone scintigraphy (d–f). (a) Plain abdominal CT scan. Arrows indicate two enlarged ovarian tumors with uneven density at two sides of the uterus. (b) Enhancement phase of full abdominal CT. The boundary and solid portion of the two ovarian tumors are enhanced in the arterial phase. (c) Bone window showed a discontinuous border and soft tissue density mass in front of the sacral promontory, indicating lesion invasion. (d–f) Radionuclide bone scintigraphy also showed focal radionuclide concentration of the first sacrum at transverse and median sagittal and coronal sections.

In December 2018, the patient complained of abdominal distension without any inducement. Ultrasound examination showed bilateral heterogeneous ovarian masses, 118 mm × 85 mm on the left and 97 mm × 79 mm on the right. Color Doppler flow imaging showed blood flow signals in both lesions. A whole abdomen CT revealed a round uneven density mass at each side of the uterus, of sizes 83 mm × 88 mm × 111 mm and 95 mm × 94 mm × 128 mm, respectively. The border and the solid part of the two lesions were enhanced slightly during arterial phase. In addition, a sheet-like mass with soft tissue density was detected in front of the sacral promontory, in which a low-density area was visible. Radionuclide bone scintigraphy showed focal radionuclide concentration of the first sacrum (Figure 1). The serum CA125 was 400.30 U/mL (normal, 0–35 U/mL); CA199 and neuron-specific enolase were normal.

The patient underwent an exploratory laparotomy. Each ovary had been replaced by an off-white solid tumor. The bilateral fallopian tubes and ovaries remained unremarkable while the uterus was congested and swollen, with abnormal anatomy. A palliative surgery was finally performed to resect the tumors and relieve symptoms of intestinal compression. Considering the possibility that the pelvic masses derived from the SCLC, total abdominal hysterectomy and bilateral salpingo-oophorectomy were performed.

**Figure 2:**
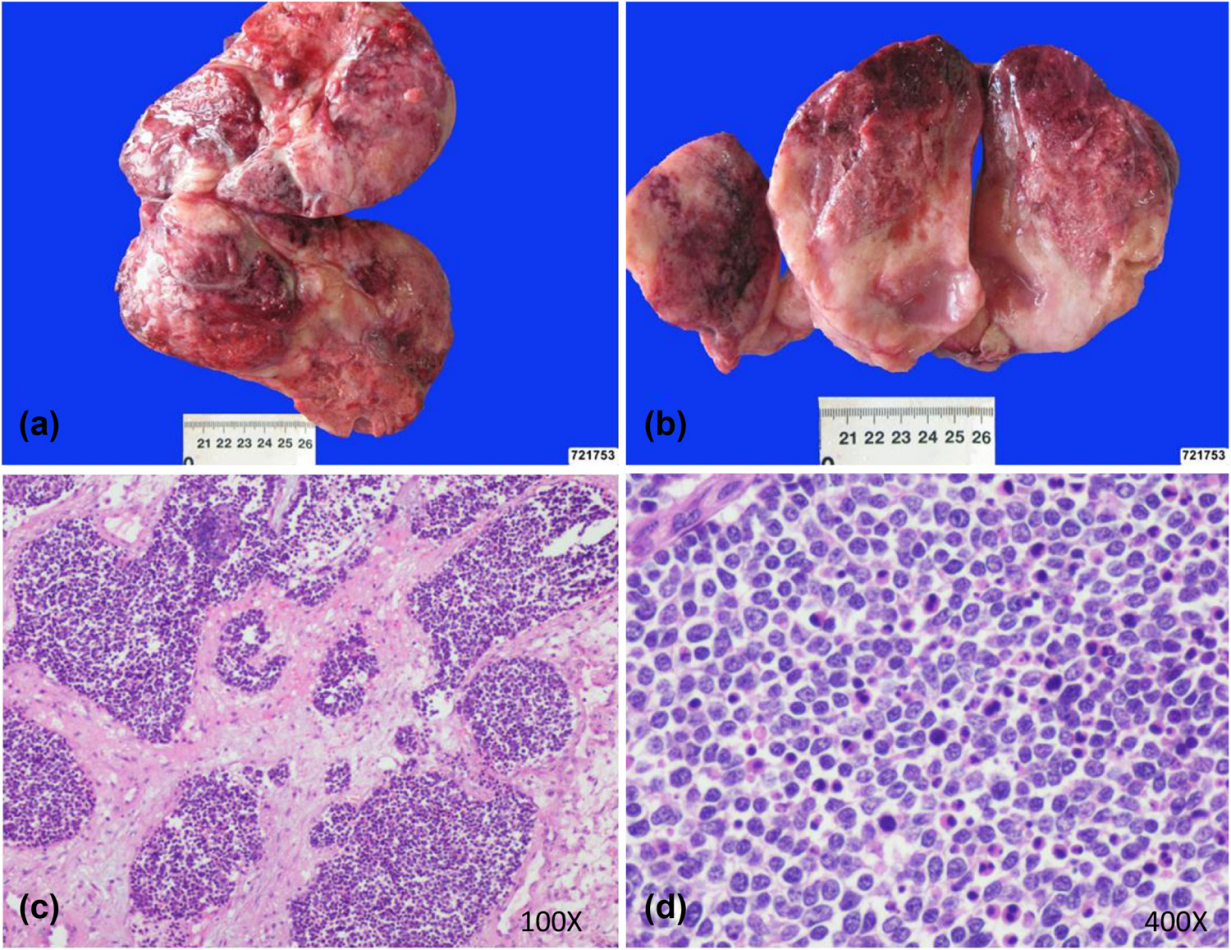
Bilateral ovarian masses (a and b) and their microscopic appearance (c and d). Gross appearance of the ovarian tumors. (a) Left ovarian tumor showing a solid mass. (b) Right ovarian tumor showing a solid mass with apparent necrosis. (c and d) Tissues stained with hematoxylin and eosin. (c) Tumor growth was in sheets and nests in which spotted and focal necrosis were observed (100×). (d) Tumor cells were mainly small, with scant cytoplasm; round to spindle-shaped hyperchromatic nuclei with inconspicuous nucleoli (400×).

**Figure 3:**
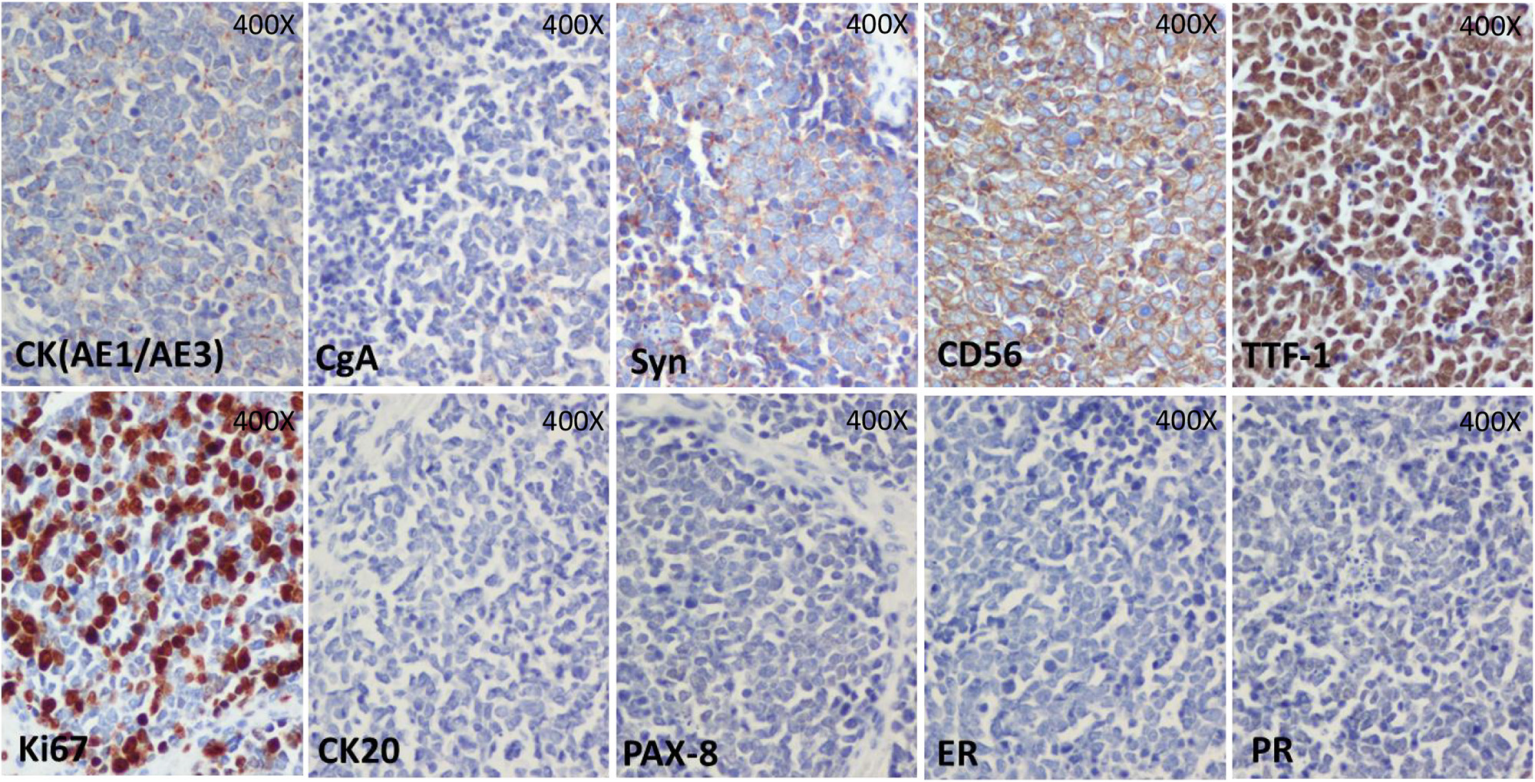
Immunohistochemical staining of tumor tissue. All original magnifications, 400×. Top row, left to right: CK(AE1/AE3), with dot-like pattern (×400); CgA, tumor cells were negative for CgA; Syn, tumor cells were weakly positive for synaptophysin; CD56, tumor cells were positive for CD56; TTF-1, tumor cells were positive for TTF-1. Bottom row, left to right: Ki67, tumor cells were positive for Ki67 with a positive rate of 60%; CK20, tumor cells were negative for cytokeratin 20. PAX-8, tumor cells were negative for PAX-8; ER, tumor cells were negative for estrogen receptor; PR, tumor cells were negative for progesterone receptor.

Histologically, ovarian tumor cells were arranged in sheets and nests, and spotted and focal necrosis were observed. Most of the tumor cells were small- or medium-sized with scant cytoplasm, and round or spindle-shaped hyperchromatic nuclei with inconspicuous nucleoli (Figure 2). Immunohistochemical staining showed the following: CK (AE1/AE3) (focal positive), CgA (–), synaptophysin (weak +), CD56 (+), TTF-1 (+), Ki67 (60%), cytokeratin 20 (–), paired box-8 (PAX-8) (–), estrogen receptor (–), and progesterone receptor (–) (Figure 3).

The patient received local irradiation at a dose of 50 Gy in 25 fractions, combined with chemotherapy comprising bevacizumab and irinotecan, 1 month after the operation. During the treatment, a large amount of ascites occurred again, indicating that the current treatment did not effectively control the rapid progress of the tumor. Unfortunately, the patient died of systemic organ failure in May 2019.


**Informed consent:** Informed consent has been obtained from all individuals included in this study.
**Ethical approval:** The research related to human use has been complied with all the relevant national regulations, institutional policies and in accordance with the tenets of the Helsinki Declaration, and has been approved by the Jilin University Experimental Bioethics Committee (Changchun, China).

## 3 Discussion

With the advent of the era of immunotherapy, in April 2022, Phase III CASPIAN study confirmed that on the basis of EP regimen, although combined with durvalumab could only extend the median overall survival of patients with extensive SCLC by 2.4 months, the 3-year survival rate could be significantly improved, showing a “long tail effect of immunotherapy.” It is suggested that patients with extensive SCLC still have the hope of long-term survival [4]. Antibody-coupled drugs (ADCs) have shown remarkable antitumor activity in breast cancer and other fields. In SCLC, ADCs such as anti-DLL3, anti-CD56, anti-TROP2, and anti-CD276 are being evaluated for clinical activity. Data from a Phase I clinical trial were presented at the 2021 ESMO meeting, which evaluated the efficacy and toxicity of anti-CD276 ADC (DS-7300) in multiple solid tumors and found that it had some clinical activity in SCLC patients [3]. In August 2020, Clinical Cancer Research released consensus recommendations for surveillance and treatment developed by the International SCCOHT Consortium. This article provides an overview of the genetic and clinical characteristics of SCCOHT, provides up-to-date information on new therapeutic targets, and makes recommendations for surveillance and treatment [6]. With the breakthrough of immunotherapy and targeted therapy in recent years, there are more and more potential therapeutic drugs and clinical trials for ovarian SCLC. However, the rare nature of ovarian SCLC poses a challenge for diagnosis and treatment. Herein, we report a case of ovarian metastasis from SCLC. We hope to combine chemotherapy with targeted therapy or immunotherapy in the future. By accumulating more samples, the factors affecting prognosis can be obtained, and the diagnosis of primary tumor and metastatic tumor can be clarified, so that more detailed treatment plans can be formulated to improve the survival rate, prolong the survival time, and improve the prognosis.

Carcinoma of the ovary due to metastasis from a distant source is very rare, accounting for only 6–28% of all ovarian malignant carcinomas [7]. Of these, the stomach is the most common extra-genital origin (60–80%) [8]. SCLC metastasizing to ovary is extremely rare and only a few cases have been reported. SCCOs can be primary or due to metastasis, and both have similar histologic features, making them difficult to differentiate and diagnose. In the present case, in order to direct the proper therapy it was important to distinguish whether the original site of carcinoma was ovary or lung.

Primary SCCO may be classified as either hypercalcemic type (SCCOHT) or pulmonary type (SCCOPT). The true origins of either remain controversial. Young et al. [9] proposed that primary SCCO originates in ovarian epithelium, while according to Ulbright et al. [10], it originates from germ cells.

SCCOHT, first reported in 1982 [11], usually occurs in adolescents and young women with an average age of 22 [12]. The most characteristic clinical sign of SCCOHT is hypercalcemia. About two-thirds of patients with SCCOHT have elevated blood calcium, only 10% of which have clinical manifestations of hypercalcemia (i.e., weakness, difficulty concentrating, confusion, stupor, and coma) [9]. The blood-calcium levels of patients with SCCOHT always range from 2.43 to 4.80 mmol/L (average, 3.68 mmol/L) and the blood calcium concentration can subside after surgery [13].

Microscopically, the SCCOHT tumor cells are arranged in nests, islands, or cords; have scant cytoplasm; small, round, or ovoid nuclei of hyper chromatin; and few individual nucleoli can be observed. Eighty percent of tumors are characterized by follicle-like space with eosinophilic luminal content. Large eosinophilic cells with abundant cytoplasm and cavitation nucleus are visible in 50% of cases [14]. The immunohistochemical staining of SCCOHT tumor cells for the following are usually positive: P53, epithelial membrane antigen, CK (AE1/AE3), calretinin, Wilms’ tumor 1 (WT1), and CD10. Immunohistochemical staining outcomes for CgA, desmin, S-100, α-inhibin, and TTF1 are usually negative. Positive staining for synaptophysin and CD56 is occasionally encountered [9].

Recently it was found that mutations of the gene *SMARCA4* (SWI/SNF [switch/sucrose non-fermentable] related, matrix associated, actin-dependent regulator of chromatin, subfamily a, member 4) may be an inducement of SCCOHT [15]. Berchuck et al. [16] suggested that mutations of *SMARCA4* resulted in the loss of BRG1 protein, which led to SCCOHT oncogenesis. These mutations may be heredofamilial.

The concept of SCCOPT was first proposed in 1992 [17]. SCCOPT is even rarer than SCCOHT, and mostly occurs in peri- or postmenopausal women. It also has neuroendocrine characteristics, but without a high blood calcium concentration [18]. Histologically, the growth patterns of SCCOPT are predominantly diffuse, sometimes in nests, and with trabeculae, glandular, and rosette-like structures. The tumor cells often have round, ovoid, or slightly spindled hyperchromatic nuclei, with “salt and pepper” chromatin in molding [14]. The cytoplasm is scant. There is usually abundant mitotic activity and frequent apoptosis. The immunohistochemical staining is always positive for CD56, chromogranin, and synaptophysin. Some cases also show nuclear immunoreactivity of the TTF-1, but this result does not prove a metastasis from lung [14].

Chan et al. [19] found that only 1 of 37 primary lung tumors showed cytokeratin 20 immunoreactivity; yet punctate cytokeratin 20 positivity was characteristic of Merkel cell cancer and salivary gland small cell cancer. This suggested a means to differentiate extrapulmonary from pulmonary small cell cancer. Rund and Fischer [20] reported two cases of primary pulmonary-type small cell cancer with perinuclear dot-like cytokeratin 20 staining. Considering the previous research of Chan et al. [19], they thought that this staining pattern was essential to differentiate SCCOPT from SCCOHT, and primary SCLC metastasizing to ovary.

SCLC metastasizing to ovary has been rarely reported. Because of the difference in therapies and prognoses, it is important to distinguish metastatic from primary ovarian cancer. However, primary small cell ovarian carcinoma and SCCOPT share similar clinical features and histological characteristics. In the present case, several features led to the identification of SCCOPT, as follows. First, the patient had a history of SCLC that had been confirmed by transbronchial lung biopsy. This suggested the likely possibility that the ovarian lesions were of pulmonary origin. Second, prior to surgery the blood calcium concentration was normal, and the microscopic observation did not coincide with SCCOHT. This helped to exclude diagnosis of SCCOHT. Third, the results of the immunohistochemical staining of the bilateral ovarian lesions were almost identical to that of the transbronchial lung biopsy. This indicated an association between the ovarian tumor and the pulmonary tumor. Lastly, referring to the reports of Chan et al. [19] and Rund and Fischer [20], the negative cytokeratin 20 staining outcome suggested a pulmonary origin for the ovarian lesions. Therefore, this patient’s ovarian tumors were diagnosed as SCLC metastasizing to ovary. The immunohistochemical staining was important to the diagnosis, and the subsequent therapy given was appropriate for SCLC.

Etoposide plus cisplatin (EP) is the most commonly used initial combination chemotherapy for patients with primary SCLC only. However, there is no effective treatment for metastatic SCLC. In the present case, the patient had received the EP regimen previously and the tumor was not well controlled, since ovarian metastasis and suspected sacral metastasis had occurred. We attempted to control the rapid tumor progression using bevacizumab combined with irinotecan, but this also failed.

Oneda et al. [2] used carboplatin alone to treat the patient in their case with no signs of recurrence after 12 months of treatment, which means that their treatment achieved a better result. Moro et al. [21] used immunotherapy to treat their patient, who was still alive at the time of publication. However, these two case reports cannot fully prove the effectiveness of either carboplatin or immunotherapy alone for treatment of patients with SCLC metastasis to the ovary, as the individual patient’s condition and tumor progression too greatly influence the effect of treatment. More cases of SCLC metastasis to the ovary need to be discussed, and more effective treatment strategies require exploration.
